# A “mysterious ghost kidney stone” in an 8-year-old boy with a solitary right kidney, obstructive megaureter and ureterostomy: Questions

**DOI:** 10.1007/s00467-020-04707-z

**Published:** 2020-07-21

**Authors:** Andrzej Badeński, Omar Bjanid, Marta Badeńska, Bartosz Chmiela, Piotr Adamczyk, Grzegorz Kudela, Grzegorz Moskal, Maria Szczepańska

**Affiliations:** 1 Department of Pediatrics, Faculty of Medical Sciences in Zabrze, Medical University of Silesia in Katowice, ul. 3 Maja 13/15, 41-800 Zabrze, Poland; 2 Department of Advanced Materials and Technologies, Faculty of Materials Engineering, Silesian University of Technology, ul. Krasińskiego 8, 40-019 Katowice, Poland; 3 Department of Pediatrics, Faculty of Medical Sciences in Katowice, Medical University of Silesia, ul. Medyków 16, 40-752 Katowice, Poland; 4 Department of Paediatrics Surgery and Urology, Faculty of Medical Sciences in Katowice, Medical University of Silesia, ul. Medyków 16, 40-752 Katowice, Poland

## Case presentation

**Fig. 1:**
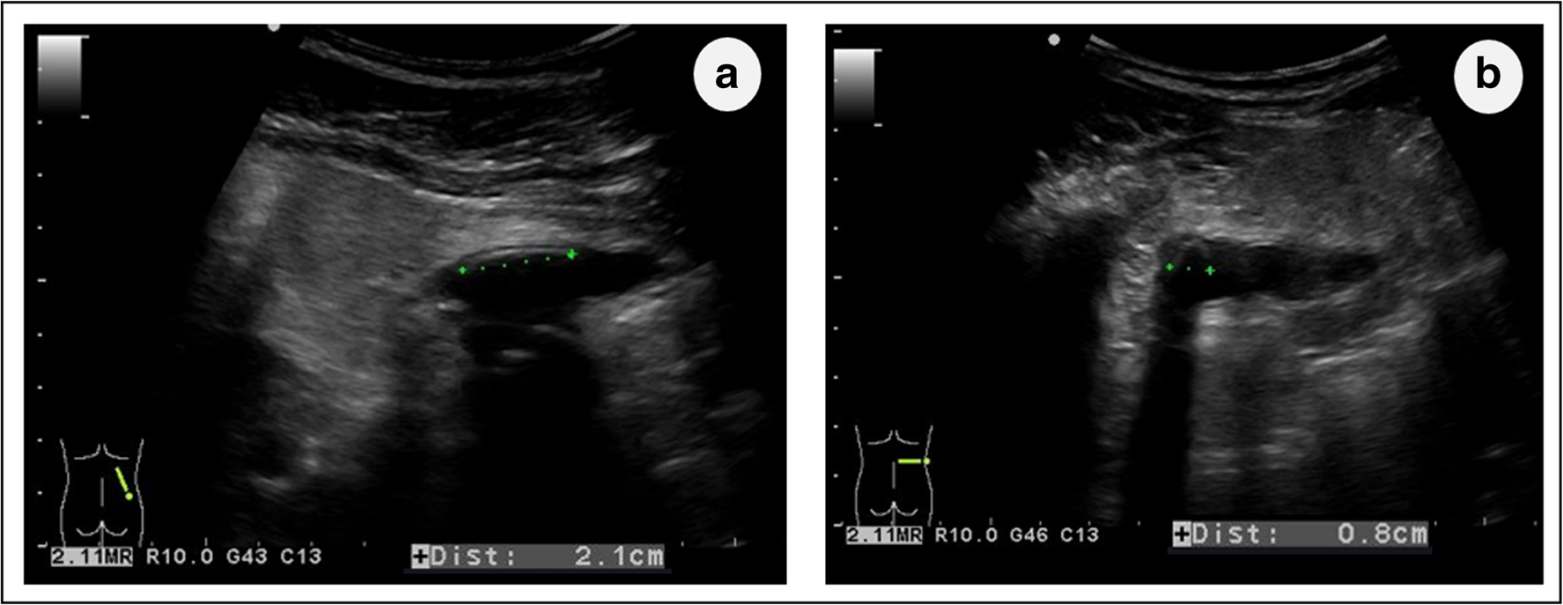
Longitudinal (**a**) and transverse (**b**) ultrasound picture of the structure in the renal pelvis

An 8-year-old Caucasian boy with a complex urinary tract anomaly and chronic kidney disease presented with a mass in the renal pelvis in ultrasound during routine check-up. The urinary tract anomaly was first revealed in the 26th week of pregnancy with bilateral hydronephrosis and possible posterior urethral valve in prenatal ultrasound. The delivery was uncomplicated with a birth weight of 3860 g and an Apgar score of 10. During the neonatal period, a voiding cystourethrography was performed and ruled out a posterior urethral valve as well as vesicoureteral reflux. Dynamic renal scintigraphy showed significant loss of the left kidney function and impaired urinary outflow from the right kidney with dilatation of the urinary tract. Due to significant hydronephrosis and residual left kidney function, the consulting urologist qualified the patient for a left side nephrectomy, which was performed without complications. A progression in the chronic kidney disease was observed with recurrent urinary tract infections during infancy. At the age of 11 months, the child was qualified for a Sober ureterostomy due to persistent right side ureterohydronephrosis. The procedure had to be performed twice, at 1-month interval, because of an early stomal stenosis. The mother of the patient maintained proper care of the ureterostomy, with daily use of urostomy bags and sealing paste, and no further complications were observed for several years. At the age of 8 years, during a routine follow-up, abdominal ultrasound showed a dilation of the right renal pelvis up to 11 mm with a slightly hyperechogenic structure within it, measuring 19 mm × 8 mm (Fig. 1), with a strong acoustic shadow but no twinkling artifact. Due to the unclear ultrasound picture, an abdominal computed tomography (CT) was perform and showed an oval structure (22 mm × 7 mm × 13 mm) with a density similar to body fat in the slightly dilated right renal pelvis. A smaller structure (< 10 mm) with similar density was located within the urinary bladder. A definitive diagnosis could not be established and the suggested differential diagnosis included, apart from atypical nephrolithiasis, a foreign body and lipoma. A first uroscopic attempt to identify the nature of the finding was also inconclusive, with no typical urolithiasis seen within the collecting system. A puzzling discrepancy arose between the abovementioned innocuous uroscopic picture and follow-up ultrasounds that persistently showed a large hyperechogenic structure with strong shadowing. After further analysis of the computed tomography examination, staghorn calculi were definitely ruled out on the basis of the low density of the lesion (minus 118–129 Hounsfield units). Lipoma still was taken into consideration. At the age of 10 years, the dimensions of the described structure in ultrasound were of 44 mm × 29 mm, with a very clear acoustic shadow and, now also, a multi-point twinkling artifact, meeting ultrasound criteria for a kidney stone. The right ureter was widened along its entire length, reaching a maximum of 21 mm between the stoma and the bladder. The child was at this point referred to an experienced pediatric urology center of reference for a second uroscopic assessment.

### Questions

1. What are common complications of high, noncontinent urine diversions?
2. What is the final diagnosis?

